# Repeatability of plantar pressure assessment during barefoot walking in people with stroke

**DOI:** 10.1186/s13047-020-00407-x

**Published:** 2020-06-29

**Authors:** A. Rogers, S. C. Morrison, T. Gorst, J. Paton, J. Freeman, J. Marsden, M. C. Cramp

**Affiliations:** 1 School of Health and Rehabilitation Sciences, Keele University, Keele, UK; 2 School of Health Sciences, University of Brighton, Darley Road, Eastbourne, BN20 7UR UK; 3 School of Health Professions, University of Plymouth, Plymouth, UK; 4 School of Allied Health Professions, University of the West of England, Bristol, UK

**Keywords:** CVA, Foot and ankle, Foot loading, Pedobarography

## Abstract

**Purpose:**

Stroke-related changes in foot structure and function affect balance and mobility and quantifying foot function following stroke could offer clinically useful information to inform rehabilitation. The aim of this work was to explore the feasibility of undertaking plantar pressure assessment during barefoot walking in people with stroke, and evaluate the repeatability of the assessment protocol and regional footprint analysis as a measure of dynamic foot characteristics.

**Materials & methods:**

Plantar pressure analysis was undertaken using a pressure platform (Tekscan HR Mat) on two test sessions, approximately two weeks apart (mean = 15.64 ± 11.64 days). Peak plantar pressure (kPa) and contact area (cm^2^) for foot regions were extracted and repeatability analysis undertaken. Descriptive evaluation of field notes and experiences of the participants was undertaken to inform the feasibility of the data collection protocol.

**Results:**

Twenty-one participants (61.8 ± 9.2 years; 11 male, 10 female; 8 right-sided, 13 left-sided stroke) were recruited and 18 returned for retesting. Full data capture was achieved from 14 participants. Peak pressure and contact area demonstrated moderate to good repeatability for at the toes (ICC 0.76 and 0.58 respectively) and good to excellent repeatability for the other foot regions (ICC ≥ 0.82).

**Conclusion:**

The protocol adopted in this study was feasible and yielded good to excellent repeatability for the foot regions, except the toes. The challenges with data collection in our study cohort could help inform future studies adopting similar protocols. This work also has relevance for use of pressure technology in clinical practice for assessing and monitoring foot function following stroke.

## Background

Stroke is a heterogenous disease [1] characterised by neurological deficit of cerebrovascular origin that persists beyond 24 h [2]. Stroke is the third leading cause of disability [3] and a wide range of impairments affecting body systems are known to occur. Complex neuromusculoskeletal factors such as muscle weakness [3], reduced joint range of motion [4], altered muscle tone [3, 5], altered foot biomechanics [6], sensory and proprioceptive deficits [3, 7, 8], movement stiffness [9] and foot problems [7, 9, 10] are commonly reported and can have considerable impact on balance, mobility and physical function. Coupled with this, localised issues affecting skin integrity such as chronic oedema, ulcer formation, hyperkeratosis, skin atrophy and foot deformity are also reported [7, 11]. Recent attempts to characterise the foot in stroke survivors reported asymmetrical foot-type(s), with both supinated (16%) and pronated (13%) foot-types observed [12]. This work reported no significant relationship between foot abnormalities and spasticity or weakness but changes in foot types were reported to be more frequent in those whose mobility was limited to indoor walking (*p* = 0.01), as opposed to community ambulation, with asymmetrical foot type more likely in household walkers (*p* = 0.003–0.038, odds ratio = 0.06–1.16). These findings illustrate a potential link between foot-specific changes and overall mobility following stroke. Despite this existing work, the impact of stroke on the foot and ankle remains poorly understood and dynamic function difficult to measure.

Plantar pressure assessment is a quantitative approach to evaluate plantar loading as an indicator of biomechanical function of the foot [13]. As with many assessment tools, feasible and repeatable protocols that can be implemented in clinical practice are essential to collect robust data to inform clinical decision making. Plantar pressure assessment has advanced understanding of the foot in healthy older populations [14–16], in people with diabetes [17, 18] and rheumatoid arthritis [19, 20]. Earlier work with a neurological population [21] evaluated dynamic plantar pressures in people with spastic and non-spastic hemiparesis of mixed aetiology. This study reported lower peak pressures under the foot regions and discussed the effects of spasticity on loading characteristics. Similarly, work looking at foot loading patterns in stroke participants reported differences in foot loading characteristics across plantar regions [22], although deficits were reported to reflect differences in walking speed. Recent work by Forghany et al [23] reported differences in plantar pressure distribution in the affected feet of stroke participants and reported that abnormal plantar pressures contributed to limited functional mobility. Based upon this existing work, there are clear benefits to plantar pressure assessment in clinical practice but reasons for the changes in foot loading characteristics are yet to be fully elucidated.

Inconsistencies with data reporting and choice of variables in plantar pressure studies have underpinned concerns about measurement protocols for pressure assessment in clinical populations, with calls to standardise data collection protocols [13, 24]. Plantar pressure measurement for clinical and research purposes requires adherence to gait protocols, but these can be difficult to implement when working with neurological populations. Impairments associated with stroke such as reduced muscle strength, impaired balance, gait changes, and cognitive impairment all impact on walking and make adherence to protocols challenging. Given the paucity of work in this field, and our limited understanding of the biomechanical profile of foot and ankle function following stroke, further work is needed to define suitable clinical assessment protocols for evaluating the foot. Establishing the repeatability of plantar pressure assessment in this population will help better define assessment measures and help inform whether plantar pressure assessment is an appropriate clinical tool for use in clinical assessment. The aim of this work was to explore the feasibility of undertaking plantar pressure assessment during barefoot walking in people with stroke and evaluate the repeatability of the assessment protocol and regional footprint analysis as a measure of dynamic foot characteristics.

## Methods

Ethical approval was granted by the host institution Research Ethics Committee and NHS Research Ethics Committee (13/SW/0302).

### Study design

A test retest study design was conducted with a group of stroke participants who were tested on two separate occasions approximately 2 weeks apart. Feasibility of data collection methods (the protocol) were determined through observation and evaluation of experimental issues experienced during the testing procedure. This included the time taken to complete testing, the number of walk trials required to gain three complete datasets, and anecdotal patient and researcher field-notes of experimental issues and difficulties in completing foot loading trials. Test-retest repeatability for peak plantar pressure and contact area across four plantar foot regions was undertaken.

### Participants

Twenty-one people who had a stroke were recruited from local stroke groups. All participants were deemed to have mental capacity and provided informed consent. The capacity of participants was determined in line with the key principles of the Mental Capacity Act [25]. Participants were recruited if they were ≥ 3 months post stroke, able to walk 10 m independently with or without a walking aid, had no other co-existing neurological condition such as Parkinson’s disease or any pathology affecting foot structure.

### Data collection

All testing was conducted in the Human Movement Laboratory at the host institution. All data was collected by one researcher who had greater than 3 years’ experience of working within the clinical setting with people with neurological deficits. Participants were asked by the researcher to self-report their typical walking ability adapted from the Functional Ambulatory Categories (FAC) [26]. The testing procedures were then explained and prior to data capture, all participants were asked to remove their socks and shoes and the system was calibrated for each individual using the ‘*step*’ calibration in accordance with the manufacturer’s protocol [27]. This required the participants to step onto the mat, during which time the sensors were calibrated to body weight. After piloting both a two-step and a mid-gait protocol, the two-step protocol [28] was chosen for data collection. This was considered more appropriate for those with mobility difficulties. The two-step method is reported as being as reliable as the mid-gait method and requires less trials [28, 29]. This approach has been used in both research and clinical settings [28, 30] and readers are referred to these studies for an overview of the different gait protocols.

Each participant was asked to walk across the mat at a self-selected comfortable walking speed. Three walking trials were planned to be recorded for each participant. A complete foot fall onto the mat was required for each walking trial. A short rest period between each trial was offered and those susceptible to fatigue were allowed to sit and rest as appropriate. Data was collected from the most affected side of the stroke participants. Data was collected on two test sessions, for the stroke group, approximately 2 weeks apart (mean = 15.64 ± 11.64 days). Testing was conducted over the summer months and this resulted in delay with organizing repeat test sessions because of pre-arranged participant vacation plans.

### Instrumentation

Plantar pressures were recorded during level walking using a high-resolution plantar pressure mat (TekScan™ South Boston, USA) (v6.7x). The system had a sensor spatial resolution of 4 sensels™/cm^2^, a sensor area of 0.48 × 0.51 m and a total number of 8448 sensors. Data was sampled at 50 Hz.

Geometric regional analysis of the plantar footprint was used to divide the foot into regions of interest. Four regions of interest were identified: the rear-foot (RF), mid-foot (MF), forefoot (FF), and toes (representing the hallux and lesser toes). Regions were derived from the total foot length (posterior mid-heel to distal point of second metatarsal head) with the foot divided into three plantar regions which were equal thirds of the total length. The toes were masked separately based on their area. The mean value of the three trials for the following two variables were extracted for analysis: [1] peak plantar pressure (kPa) - defined as maximal pressure recorded during stance through one region of the foot [2]; contact area (cm^2^) - defined as the total area in contact with the mat during stance within a specific foot region. One person (AR) undertook all masking of the data in line with the pre-specified protocol.

### Data analyses

A descriptive approach to the feasibility of the protocol was undertaken based on researcher field notes and participant feedback of the experimental procedure; the data was reported in narrative form. All plantar pressure data was extracted and managed using Microsoft® Excel and analysed using SPSS (version 22.0). Prior to analysis, the data was determined to be normally distributed, using the Shapiro-Wilks test for normality. Demographics were reported using mean and standard deviation and counts. Test-retest repeatability for peak plantar pressure and contact area was explored on the most affected side in four plantar foot regions using intra class correlation coefficients (ICCs), model [1, 3]. ICCs were reported as poor (ICC < 0.5), moderate (ICC 0.5 to 0.75), good (> 0.75) or excellent (ICC > 0.90) [31]. Standard error of the measurement (SEM), mean difference, limits of agreement and Bland Altman plots were evaluated as these are deemed appropriate for test-retest analysis [31, 32].

## Results

Complete data was collected on 14 of the 21 recruited participants. Demographics for those recruited and those with complete data are provided in Table 1. All participants were independently walking over 10 m (with or without use of and aid). Five used walking sticks indoors. No one used orthotics during the experimental procedure. Using the Functional Ambulation Category (FAC), 21% were rated as FAC 4 (independent ambulatory - level surfaces only); and 79% as FAC 5 (independent ambulatory). To obtain three complete footfalls for analysis, the participants typically required seven walking trials with an average of 15 ± 5 (range 7–32) foot contacts on the mat.

**Table 1:** Demographics of the participants (mean ± standard deviation is indicated)

	All Stroke participants (n = 21)	Stroke participants (used in data analysis) (n = 14)
Mean age (SD) (years)	61.8 (9.2)	60.8 (9.2)
Mean weight (SD) (Kg)	78.6 (14.2)	77.7 (13.3)
Height (SD) (m)	1.66 (0.11)	1.67 (0.13)
Gender	11 males; 10 females	8 males; 6 females
Time since stroke (SD) (months)	67.5 (56.3)	60.1 (56.1)
Side affected by stroke/non dominant side	8 right; 13 left	8 right; 6 left
Foot length (SD) (cm)	N/A	Least affected 20.15 (1.13) Most affected 19.75 (1.11)

Incomplete data collection from seven participants (33%) occurred due to challenges with footfall onto the pressure-platform, with two participants demonstrating overlapping foot loads during one data collection event, meaning data could not be extracted. Five had no retest data; three did not attend for follow up testing and two had difficulty with appropriate foot placement on the mat.

Data collection sessions required up to 15 min. Detailed, and repeated, verbal instructions about the testing procedure were necessary. Securing the required three footfalls directly on the pressure platform was an ongoing challenge and one participant struggled to ambulate over the mat without shoes, possibly due to the loss of shoe support around the foot. Fatigue and the requirement to rest between trials was also an important consideration during data collection. Repeat trials were necessary to secure a complete foot fall in 50% of the participants. Participants did not raise any concerns with the length of the data collection session. As expected, those who required a high number of gait trials became more fatigued and they reported finding the session easier on the second visit. The data collection process required that individual foot masks for each trial were created increasing data extraction time frames (taking approximately 15 min to complete).

**Fig. 1:**
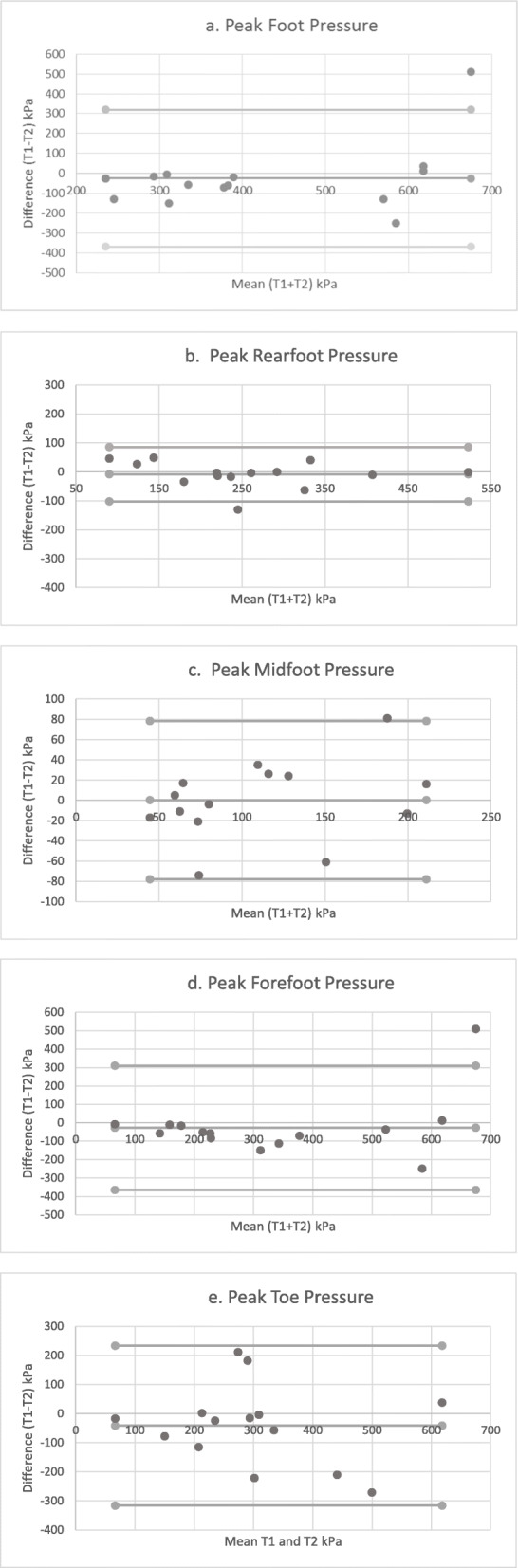
a-e Bland-Altman plots demonstrating agreement for peak pressure on two test occasions

**Fig. 2:**
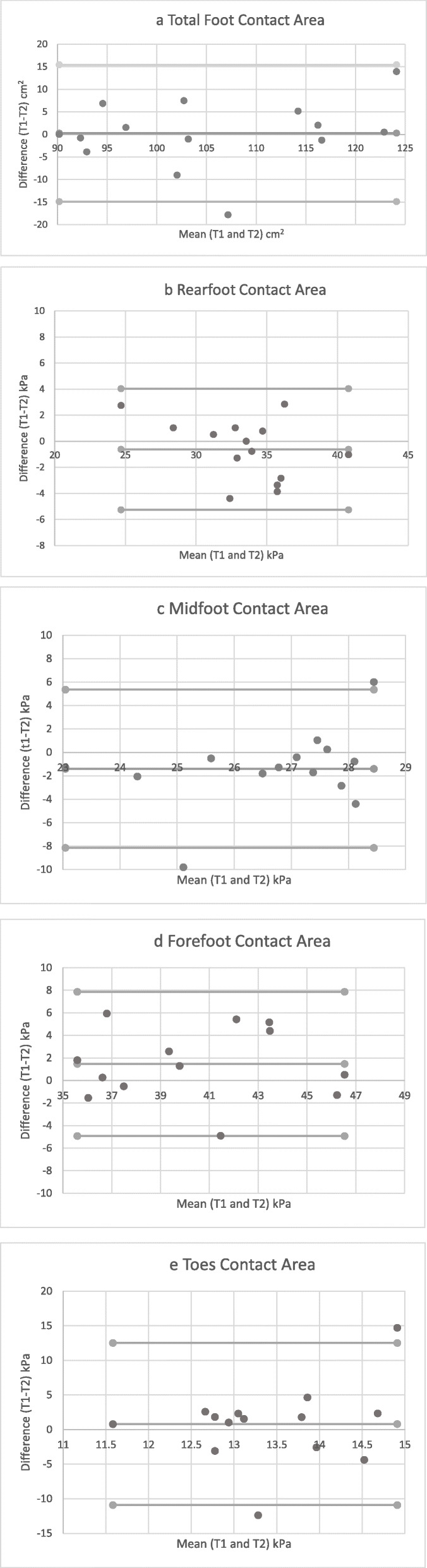
a-e Bland-Altman plots demonstrating agreement for contact area on two test occasions

Table 2 details the data for test-retest repeatability. Peak pressure (PP) and contact area (CA) demonstrated good to excellent repeatability in the four-region model for foot regions (ICC ≥ 0.82), except for the toes. The RF and MF regions demonstrated the highest repeatability (ICC 0.96 and 0.89 respectively) for peak pressures and the toe region the lowest for peak pressure and contact area (ICC 0.76 and 0.58 respectively). Whilst these results are encouraging, confidence intervals for most ICCs were large. Figures 1a - 1e and 2a - 2e show Bland Altman plots; whilst outliers are observable on the plots, the majority of the data points demonstrate an even spread around and along the mean, indicating good reliability.

**Table 2:** Test-retest repeatability for peak pressure and contact area in stroke participants

	Test 1 Mean (SD)	Test 2 Mean (SD)	Test Mean (SD)	Mean Difference	ICC (95% CI)	SEM
Peak Pressure (kPa)						
RF	253.07 (112.61)	261.07 (122.26)	257.07 (115.07)	−8.00	0.96 (0.89–0.99)	23.01
MF	111.57 (64.94)	111.35 (53.25)	111.46 (58.27)	0.21	0.89 (0.64–0.96)	19.33
FF	318.21 (238.04)	345.50 (1185.13)	331.86 (209.71)	−27.29	0.82 (0.44–0.94)	88.97
Toes	281.71 (143.98)	323.00 (169.55)	302.36 (155.77)	−41.29	0.76 (0.29–0.92)	76.31
Foot Peak Pressure	318.21 (238.04)	345.50 (1185.13)	331.86 (209.71)	−27.29	0.82 (0.44–0.94)	88.97
Contact Area (cm^2^)						
RF	33.20 (3.50)	33.81 (4.43)	33.51 (3.93)	−0.62	0.91(0.72–0.97)	1.18
MF	27.41 (10.18)	43.51 (11.84)	28.10 (10.86)	−1.40	0.98 (0.91–0.99)	1.54
FF	47.26 (6.25)	45.20 (5.62)	46.23 (4.43)	2.06	0.86 (0.56–0.96)	1.66
Toes	15.08 (5.46)	14.29 (5.17)	14.68 (5.23)	0.79	0.58 (−0.37–0.87)	3.39
Foot Contact Area	123.02 (17.36)	122.75 (16.72)	122.89 (16.72)	0.27	0.95 (0.85–0.98)	3.74

*RF* rear foot, *MF* Midfoot, *FF* forefoot, *SD* standard deviation, *SEM* standard error of measurement

## Discussion

The aim of this work was to explore the feasibility of undertaking plantar pressure assessment during barefoot walking in people with stroke and evaluate the repeatability of the assessment protocol and regional footprint analysis. The findings demonstrated that the four-region mask was feasible to implement in this sample and yielded data with moderate to excellent repeatability. This work has also identified some of the challenges with collecting plantar pressure data in stroke participants.

Given the challenges of capturing robust gait data in neurological populations, we evaluated the feasibility of plantar pressure assessment in people with stroke. Data from seven participants was lost during the study due to difficulties with the data collection process, which affected foot loading on the pressure plate and meant retest data was not practicable to collect and/or mobilisation was not achieved over the mat. In our participants, there did not appear to be any association between mobility status and the ability to capture data. Further, we did not encounter any safety issues (such as instability on the mat) during our data collection.

Step protocols are an important consideration in plantar pressure research [13, 24]. The two-step protocol in this study was found to be acceptable for these participants with varying mobility (with or without aid) and fatigue levels. Our approach helped ensure that adequate foot loading was achieved for data extraction for most of our participants, with fewer instances of multiple partial foot loads which we encountered during preliminary trials with the mid-gait protocol. We used three trials for each participant and, whilst several repeat footfalls were required to collect this number, on average 15, our approach helped ensure that adequate foot loading was achieved for data extraction for two thirds of our stroke participants. It required up to 15 min for data capture which may be practical in a research context but may not transfer well to clinical settings.

Previous authors have commented that developing participants’ confidence with walking across the pressure mat is important [33]. We also found this to be the case, with greater explanation of the testing procedure required for the participants. This has implications, both with regard to training of testers (or clinicians), and testing time. Repeat trials were required and this has potential implications given the problems with fatigue that are commonly experienced by stroke survivors [34]. Whilst not standardised in this study, protocols need to allow rest periods to ameliorate the effects of fatigue.

Test-retest repeatability of plantar pressure assessment in our participants was good to excellent for the four plantar foot regions (ICC ≥ 0.76) for peak pressures and moderate to good for contact area (ICC ≥ 0.58). Whilst the ICC values are promising, our sample was small and the 95% confidence intervals were broad, thus introducing some caution with this interpretation. These data suggest that pressure assessment is repeatable, which provides some support for the role of pressure assessment to advance understanding of foot biomechanics in people with stroke. To date, we are not aware of any studies which have reported repeatability of pressure data in stroke participants and therefore, further comparison is restricted to healthy older adults. Our findings were broadly in agreement with previous work in an elderly population [35]. This study used a 7-region foot model and, despite this difference, mean reported repeatability coefficients for the heel (ICC 0.87, 0.83–0.95), mid-foot (ICC 0.95, 0.45–0.84) were similar to the data reported in this study. Despite differences in the pressure measurement system, our results support the opinion of Gurney et al. [36] that areas of lower loading, such as the toe region, represent less reliable foot sections.

It is important for clinicians and researchers to consider the regions of interest and whether these can be captured. The toes and mid-foot were determined to be less repeatable foot regions in our study. Further consideration of these regions is important due to the changes in foot structure [12] and increased incidence of lesser toe deformity (i.e. clawing of the lesser toes and hitch-hikers toe) [11, 37]. This has been shown to predict risk of ulceration in people with diabetes [18], and thus may also be of relevance to stroke survivors. Given our findings, further consideration of the potential benefits of in-shoe pressure assessment is warranted.

The results of this study should be considered within the context of the study limitations. Due to the complexities of the sequelae following stroke, we were not able to control for all anthropometric, medical and biomechanical variables which may have impacted on the pressure data collected in this study such as walking speed, and stride length.

## Conclusion

Our plantar pressure assessment protocol was determined to be feasible and yielded repeatable plantar pressure data for the foot regions (except the toes) in our sample of stroke participants; we were able to collect completed data in 67% of our participants. Collection of plantar pressure data is not without challenges and issues with step protocol and complete foot fall on the pressure platform were identified in this study. Use of the four-region foot mask yielded data with good to excellent repeatability for RF, MF and FF regions for both contact area and peak pressure. The protocol may hold relevance for clinical practice as the clinical assessment of the foot in stroke survivors is an important component of stroke rehabilitation.

## Data Availability

The datasets used and/or analysed during the current study are available from the first author on reasonable request.
